# The Science of Lie Detection by Verbal Cues: What Are the Prospects for Its Practical Applicability?

**DOI:** 10.3389/fpsyg.2022.835285

**Published:** 2022-04-05

**Authors:** Tim Brennen, Svein Magnussen

**Affiliations:** Department of Psychology, University of Oslo, Oslo, Norway

**Keywords:** verbal lie detection, lie detection accuracy, strategic use of evidence, automated lie detection, criterion based content analysis

## Abstract

There is agreement among researchers that no simple verbal cues to deception detectable by humans have been demonstrated. This paper examines the evidence for the most prominent current methods, critically considers the prevailing research strategy, proposes a taxonomy of lie detection methods and concludes that two common types of approach are unlikely to succeed. An approach to lie detection is advocated that derives both from psychological science and common sense: When an interviewee produces a statement that contradicts either a previous statement by the same person or other information the authorities have, it will in many cases be obvious to interviewer and interviewee that at least one of the statements is a lie and at the very least the credibility of the witness is reduced. The literature on Strategic Use of Evidence shows that features of interviews that foster such revelatory and self-trapping situations have been established to be a free account and the introduction of independent information late and gradually into the proceedings, and tactics based on these characteristics constitute the best current general advice for practitioners. If any other approach 1 day challenges this status quo, it is likely to be highly efficient automated systems.

## Introduction

If there were highly reliable easily accessible cues to lying, many everyday challenges, including in police interview rooms and courtrooms, would be trivial to resolve. For this reason, there has been a substantial research effort aimed at uncovering such cues and developing appropriate methods. Some sober, solid reviews of the literature earlier this century have shown that cues to deception are, at best, faint, and typically lead to people scoring just above chance (DePaulo et al., 2003; Bond and DePaulo, 2006), and that even when one combines cues the theoretically possible detection rate does not exceed 70% (Hartwig and Bond, 2014). In the light of more recent work, there does not seem to be reason to dilute the negative conclusion. On the contrary: Luke (2019) has shown that the cues to detection are in fact likely to be even weaker than DePaulo et al. (2003) concluded. Using Montecarlo simulations, he showed that assuming a slight publication bias in favour of significant effects, the empirical database is indistinguishable from one we would expect to observe should humans in fact be unable to use verbal or nonverbal cues to spot a lie at all. In addition, in his analysis, the more a cue has been studied the less evidence there is that it is useful for detecting lies, and it also highlighted how the highly flexible definitions and ways of coding cues used in the studies will have tended to increase the estimates of their utility. Some researchers no longer even regard nonverbal behaviour as a promising *possible* source of such reliable and strong signs of lying (Brennen and Magnussen, 2020), whereas others maintain that future work may yet reveal such cues (Vrij et al., 2019), but there appears to be agreement that no such cues exist *currently*, that is, after more than 50 years of research and several hundred empirical studies (Vrij et al., 2019). Researchers also agree that it is unfortunate that the lack of generally useful nonverbal cues to deception is underappreciated among laypeople and ignored by an ever-present charlatanical ‘lie detection industry’ that sells courses, seminars and consultant services pushing methods that do not have empirical backing (Denault et al., 2020). There is no longer optimism in the research community that there is a ‘silver bullet’ of lie detection, that a clearly defined physical cue that consistently reveals lies will be uncovered, be it a simple visible cue like face touching or gaze aversion, or more subtle, like microexpressions (Ekman, 1985; Burgoon, 2018). Generally, approaches based on putative differences in anxiety and physiological activation between liars and non-liars are accepted to be unpromising and are no longer the subject of much scientific interest. Iacono and Ben-Shakhar (2019) reviewed evidence for the polygraph, concluding both that the evidence is weak and that its proponents consistently misinterpret this evidence in a favourable direction. Davis (2021) reviewed the evidence for other objective physiological and neuroscientific technologies that are being developed to detect lies and concluded that there are none with reliable and valid results.

Instead, the focus has mainly turned to verbal cues to deception. There is a plausible scenario as to why verbal cues may be a more promising domain to find differences between liars and truth-tellers: While these groups have similar strategies when it comes to nonverbal behaviour (keep nervousness to a minimum) they appear to have different strategic incentives in the verbal domain: Truth-tellers can just interrogate their memories and report all they can recall, whereas liars know that they may be caught out if they give too many details, and increasingly complexity of an account imposes a considerable memory burden consisting of relatively poorly-encoded elements that also may lead to them being trapped by inconsistent accounts. Researchers have tried to exploit this difference in cognitive demands on truth-tellers and liars with a variety of methods.

There are many contexts in which the reliable detection of verbal lies would be useful: In everyday life, minor disagreements could be easily resolved if there were good cues to point out which party to a conflict was being deceptive. In situations where a crime is thought to have been committed, or about to be committed, even more is at stake, for instance for law enforcement determining whether a suspect or victim or witness is being truthful, or detecting who in a crowd or queue has malignant intent. The research effort over the past decades has moved from a search for general purpose lie detection tools that might be usable in a very wide range of contexts to those focussing more on the police interview situation, or as an aid to courts where decisions on who is being truthful have to be made. In this paper, we will look at the progress made in diverse branches of research aimed at verbal lie detection. Reflecting how the field has developed, these are methods that have mainly been aimed at contexts like the police interview or the courtroom where the question of interest will often be specific rather than general, ‘Is this person lying?’. We also include recent technological approaches that may provide a route to mass screening to spot those who intend to commit a crime. In this paper, our aim is to look at the progress made by these branches of research and evaluate whether there is empirical justification to adjust the recommendations of best practice.

## Current Best Scientific Advice to Practical Situations Requiring Deception Detection

What would the current ‘gold standard’ advice from a balanced reading of the scientific literature on lie detection be? If instantaneous and reliable lie detection is beyond human capabilities, then the activity becomes less about seeing through deception with psychological X-ray sspecs and more about demonstrating possible lies through a clash with the established facts or documents of the case. Alternatively, and with a sensitivity to the limits of the precision of human autobiographical memory, simply documenting that the person has given contradictory accounts on different occasions would suggest that at least one is incorrect, inherently reducing the person’s credibility. These intuitive rules of thumb can be taken as the baseline of lie detection, and below we ask whether the post-DePaulo research has given practitioners any methods, strategies, tricks, or tips that are more useful and precise than these ground rules. Keep in mind that the task of the police interviewer is usually focused on the lie status of one person’s account about a single event, often from a single interview. One needs a relatively definitive answer to the question of whether this particular person is lying now, not a vague indication that s/he is lying.

Vrij et al. (2018) listed four lie detection methods based on interviews that have best documentation or the highest potential, and here, we discuss those four, plus Criterion-Based Content Analysis because of its widespread use. We categorise them according to whether they analyse naturalistic statements, manipulate the production of statements or require independent information and finally consider lie detection by machine.

## Systematic Analysis of Statements

Some methods that have been used to investigate verbal cues operate without intervening in the information gathering process and without the need for extraneous information about the incident or even the person is talking about. This is analogous to much of the work on nonverbal cues. One records the verbal behaviour naturally produced by truth-tellers and liars and the methods are applied to the data to analyse whether there are reliable differences.

### Criterion-Based Content Analysis

There are methods based on a general assumption that an account based on an actual experienced event will draw upon richer mental representations than an account based on a fictional event, and so the former type of account will be tend to be more marked by perceptual and affective features and richness of detail. These include Reality Monitoring and Criterion-Based Content Analysis (CBCA), which differ in detail only. CBCA is deemed to be the most widely used method to detect lies across the world and is admissible in many national jurisdictions. In it, a statement is evaluated using criteria, the presence of which is taken to indicate that the account is based on true experience rather than being made up. The types of criteria include specific details, peculiarities and motivation, and examples of criteria are *Unexpected complications during the incident*, *Accurately reported details misunderstood*, *Admitting lack of memory* and *Pardoning the perpetrator*.

Many studies have investigated to what degree the criteria distinguish between truth and lies, and Oberlader et al. (2016, 2020) have performed comprehensive meta-analyses, concluding essentially positively that CBCA successfully discriminates between true and false statements, though markedly less so in the latter paper. Of course, the important question is how well it does so and Oberlader et al. (2016) provide careful statistical analyses that show the effect is medium to large. They also discuss in a nuanced way how such effects can be applied to single cases in practical contexts. On the one hand, they say the content-based techniques ‘provide substantial potential’. On the other hand, they point out how, even assuming the upper end estimate of the effect size, tuning the techniques to high rates of lie detection has the consequence of causing high rates of wrongly classifying true statements as false, and conversely, tuning the decision criterion to keep such false alarms to a minimum lead to low hit rates of detecting actual lies. For instance, a 95% hit rate for lie detection would also falsely categorise 75% of true statements as lies. And using a more conservative criterion to keep false alarms to 1% would reduce correct classifications of lies to under 9%.

One might think that such figures would suffice to show that the method should only be used with the greatest of caution and scepticism, even if used as only one of several sources of evidence to decide veracity. Oberlader et al. (2016) argue that the CBCA effect size is equivalent to that of our ability to identify faces^1^ and that that type of evidence is often used in courts. But they overlook the fact that as a source of evidence the recognition of previously unfamiliar faces is in the US heavily associated with both miscarriages of justice and similar cases that almost resulted in wrongful convictions (Gould et al., 2014), so that having the same effect size as this type of evidence is not grounds for recommending the use of CBCA in courts. Oberlader et al. (2016) argue that because no other method is demonstrably better, CBCA may be recommendable in practical contexts; however, they do not reconcile this suggestion with their sober assessment of the dangers of the high false alarm rates. As noted, Oberlader et al.’s (2020) conclusions are more reticent regarding the implementation of CBCA than the first paper.

Wojciechowski et al. (2018) performed a study that investigated whether training in CBCA and a similar technique increased truth detection. One notable feature of their article is that although they found training increased truth detection to around 70%, in the range found by many other studies, they point out that this does not show that CBCA can be used by itself to assess truth that the detection rate is far from beyond reasonable doubt and that we do not currently have such tools.

An additional problem for CBCA has been pointed out by Kleinberg et al. (2019) who show that the estimates of CBCA’s utility have been systematically overestimated due to conflating the optimal statistical discrimination that CBCA affords between truthful and deceptive statements, and actual human performance. The best guess of the magnitude of this overestimation is 12%, which reduces the assumed utility of the method from around 70 to 58%, barely above chance.

We have focused on CBCA because it is the most widely used method of its type. It seems unlikely that there would be a more positive conclusion from, for example Reality Monitoring, largely because it rests on the same assumptions as CBCA, and the available evidence, for instance from Oberlader et al. (2020), is not more positive than for CBCA. Oberlader et al. (2020) find no evidence that another method, Scientific Content Analysis, is able to distinguish true statements from false at all.

### Verifiability Analysis

Nahari et al. (2014) drew a distinction between verifiable and non-verifiable details contained within an account. For instance, if a suspect said that she was alone in her kitchen at the time of the burglary this may be difficult to verify, compared to an account from a suspect who said that she was on the number 25 bus at the time. Nahari et al. (2014) proposed a plausible cognitive scenario where liars are in a kind of catch-22 in which on the one hand they know that credible accounts contain specific details about the event, and on the other hand they also know that a sceptic or an investigator may wish to check any details that one gives in an account. This led to the predictions that the accounts of truth-tellers would contain more verifiable details, whereas the accounts of liars would contain more non-verifiable details. Some participants were instructed to provide a completely untrue statement and others a completely true statement, and Nahari et al. (2014) observed that liars’ accounts contained significantly fewer verifiable details compared to truth-tellers but that there was no difference between the groups for non-verifiable details.

Verschuere et al. (2020) reported a meta-analysis with a total of 28 comparisons between liars and truth-tellers on verifiable and non-verifiable details. It showed that the pattern of observations reported by Nahari et al. (2014) has been borne out by subsequent research, namely, that liars do not produce more non-verifiable details than truth-tellers but that truth-tellers do provide more verifiable details, with a moderate effect size. These seem like solid, replicable effects so that the question is to what extent the verifiability approach is now sufficiently documented to be transferred to real-world settings. Verschuere et al. (2020) point out that most of the studies are underpowered and that the originators of the verifiability idea, Nahari et al. (2014), were, together or separately, co-authors on many of the studies with largest effect sizes, so that it seems premature to suggest incorporation of the method in forensic settings. In addition, the verifiability approach has yet to be demonstrated in field studies. At the same time, the literature has usefully falsified the plausible and theoretically motivated idea that liars produce more *non*-verifiable statements than truth-tellers.

## Methods That Manipulate Statement Production

A second important branch investigates lie detection in more convoluted and structured verbal situations. This appears to start addressing the issue of ecological validity for which the field has previously been criticised. These include settings such as the police interview that last over time, where one can take repeated measures from the same target person and compare the target’s behaviour under different conditions, for example while answering different types of questions.

In the forensic context, lie detection becomes more a sophisticated investigation over time, where control conditions and repeated measures can be used but where the task difficulty increases due to the ample opportunity for the suspect to play complex psychological games, including convincing him- or herself of the truth of the lie, rendering detection more difficult, be it by psychological or physiological means. For this reason, the suggested methods are also necessarily more complex. Vrij and Granhag (2012) explicitly proposed this change of direction where methods be developed where the lie-catchers actively interview possible deceivers with systematic methods suited to generate obvious lies. This proposal has been called a paradigm shift (Kassin, 2012) and a considerable amount of data had been collected since then under the umbrella term of ‘cognitive’ approaches to lie detection, in contrast to the anxiety-based approach that dominated previously. In a meta-analysis that included many cognitive approaches to lie detection, the best-controlled studies showed that while untrained observers are barely better than chance at lie detection, training increases the rate to 75% (Mac Giolla and Luke, 2020). It is however also pointed out that the research as a whole shows signs of a publication bias towards positive results and that it is affected by troubling problems of design. This overall look at the field suggests that it is premature for translation to practice. Here, we look at the evidence for the utility of the two methods that manipulate statement production that Vrij et al. (2018) name as the most promising.

### Assessment Criteria Indicative of Deception

This method is based on tried-and-tested, theory-based ways of eliciting information from an interviewee, the Cognitive Interview (Fisher and Geiselman, 1992) and the Reality Interview (Colwell et al., 2007). The Cognitive Interview contains intellectually demanding components (e.g., reporting the event in different orders or from different perspectives) that, serendipitously, affect liars more than truth-tellers. A Reality Interview in addition contains Yes/No questions that also seem to disrupt liars’ cognitive strategies and facilitate detection.

There are some single studies that definitely warrant some optimism. For example, Colwell et al. (2015) trained a group of experienced US police officers for 8 h on how to interpret details of a statement with regard to truth or lie, based on scientifically sound criteria. When presented with statements to decide the truth status of, this group went from pre-training barely above chance to around 90% after.

The generalizability and replicability of the finding will have to be probed, for instance, how long does the effect of training last and does the effect found in audio statements generalise to live interviews? On the basis of these data Assessment Criteria Indicative of Deception (ACID) is currently not close to being ready for the field. Another feature of the status of ACID is that a single laboratory is the source of much of the data; before implementing widely, it would be necessary to see positive results with the method from many other laboratories too. It also looks likely that it will be possible for reasonably sophisticated suspects to take countermeasures to ACID by learning what criteria the method relies on.

### Cognitive Credibility Assessment

This method combines components that may each contribute to lie detection. These are *Imposing cognitive load*, *Asking unexpected questions* and *Encouraging interviewees to say more*. Of the three the latter appears to show most promise (Vrij and Fisher, 2016). For readily understandable reasons, liars are reluctant to give more details than necessary, because the false narrative becomes cognitively unwieldy and they may be caught in a lie. In a meta-analysis of studies that used Cognitive Credibility Assessment, Vrij et al. (2017) showed that training in it raised performance to about 70%. In other words, it would appear to pick up some lie signal but with plenty of errors too. However, the results of this meta-analysis were strongly criticised by Levine et al. (2018), who argued that Vrij et al.’s (2017, p. 7) ‘analyses confounded dependent variables, capitalised on aberrant controls, and used unreliable data to inflate support’. Their own meta-analysis of the same data showed a much smaller advantage by the cognitive approach and that the findings most supportive of Vrij et al.’s (2017) conclusions came from the least reliable data.

## Assessment of Methods That Analyse and Manipulate Statements

None of the methods that systematically analyse verbal statements or ones that manipulate their production have better than sparse support and none of them are close to being able to be recommended in practical settings. There is another methodological concern to which many of the studies reviewed here are vulnerable. The issue is that over the course of an experiment where a range of stimuli is presented, participants implicitly develop a sensitivity to the parameters of the stimulus set and that this artificially increases the apparent discrimination rate (in the current context between lies and truth), an effect well known in visual psychophysics (Lages and Treisman, 1998; Magnussen et al., 2003). In many studies covered above, participants were taught to use particular cues, for example that on average untrue accounts are shorter than true ones, and the participants will over the course of the test phase have been able to adjust their decision criterion to become more competent at distinguishing lie from truth for that particular set of stimuli or for the range of stimuli presented. The problem is that this may lead to an overestimate of the method’s real utility outside the context of the particular experiment. In practice, a lie-catcher will usually have no comparison set by which to gauge what is normal: There is one statement and one wants an answer as to whether or not it is a lie. In more colloquial terms: Is this a long piece of string? Well. In the context of these long pieces, it is short and in the context of these short ones, it is long. But if you have to judge on the basis of a single piece of string there is no aid to be had from other trials.

In order to rule out this type of intra-experiment context effect, or indeed to determine how much it may have led to overestimates of methods’ discriminative ability, a study would need to train participants in the lie detection method under consideration and then collect one decision on a single statement per participant. Regarding the existing database of studies of lie detection training, one would need to reanalyse the data looking only at each participant’s *first* decision in the test phase. This would give a truer (and perhaps lower) estimate of lie-catchers’ ability to detect lies with the method.

A similar point has been made in the commentary by Taylor, Maroño and Warmelink in Nahari et al. (2019), where they demonstrate that lie detection methods that significantly distinguish between groups of liars and truth-tellers can also have very poor ability to say whether a particular statement is true or not. As for CBCA discussed above, there is no decision criterion that satisfactorily discriminates between truthful statements and lies. Satchell (2019) shows how even statistically large effect sizes between lies and truth that one observes with a method may coexist with large overlap between the distributions. To provide a more intuitive way of telling how good a method is at categorising statements as true or false, Satchell proposes a statistic called Overlap Coefficient as an adjunct to measures of statistical difference between group means.

## A Method That Requires Independent Reliable Information

Strategic Use of Evidence (SUE) requires that the lie-catcher be in possession of reliable information about the event in question independent of, and prior to, the interview. In such cases, SUE is equivalent to an instantiation into a method of the sensible basic advice from the science outlined above. In concert with a free account, the judicious introduction of the other evidence the police possess (generally *late* in the interview, and *gradually*), SUE has been shown to lead to changes of substance in an interviewee’s statements thereby potentially trapping them in a lie or forcing them into changes of account that reduces their credibility; this seems by far the most promising human-based method, incorporating solid (and simple) psychological knowledge that can be applied in certain forensic contexts, but not others; and so it is not suited for mass checking of airport passengers for example, but for highly labour- and expertise-intensive investigations of low numbers of suspects, usually a single one (Hartwig et al., 2014). There are eight studies giving a total of 16 effect sizes.

Oleszkiewicz and Watson (2020) performed a meta-analysis of studies that vary *when* during a police interview the suspect is confronted with the available evidence. Typically in these studies, a mock crime is set up and one group of participants are instructed to transgress in some way, whereas another group performs similar but entirely legal activities. They are subsequently interviewed about their actions and all participants attempt to convince the interviewer that they are innocent of the crime, e.g., Jordan et al. (2011). Even though there are only 16 studies in the meta-analysis, it is clear that ‘guilty’ participants were caught in more lies when disclosure of information is late in the interview and gradual, in the terminology, they produced more ‘Statement-Evidence Inconsistencies’ and that this is a large effect. This approach can thus be regarded as promising, more so because several of the studies included in the meta-analyses that show positive results had police interviewers as participants. Arguing a different route to us, Vrij and Fisher (2016) also concluded that SUE is unsurpassed by alternative methods. It should be noted that ‘innocent’ participants also produce Statement-Evidence Inconsistencies so that their presence indicates a need for investigative follow-up rather than an immediate conclusion of guilt.

As noted, in order to be applied, SUE requires an investigator to have access to established facts about the case from sources other than the interviewee. This necessarily entails that SUE has a more restricted domain of application than the other methods discussed. On the other hand, Luke et al. (2018) argued that this reliance on facts of the case allows SUE to be a useful part of the dynamic investigative interview that facilitates gathering of evidence. That is consistent with Taylor et al’s commentary in Nahari et al. (2019) where they propose to reframe the field of verbal lie detection as the field of information elicitation.

Lastly, we note that a recent study investigated the ability of complex verbal analysis to distinguish between factually correct and incorrect statements for a single person in a restricted domain, namely, in Donald Trump’s tweets (Van Der Zee et al., 2021). Even with the specificity of the stimuli used, the hit rate for the analysis was only 74%. So, while the method significantly distinguishes between true and false statements, there is no reason from this study for optimism regarding the more general application of such methods to achieve forensically applicable hit rates.

## Verbal Lie Detection Using Automated Techniques

Another reaction in the research community to the lack of nonverbal cues to detection has been a move to automated detection of deception. This circumvents the apparent limitations of the human cognitive system to detect human lies and enlists the help of computational power to provide adequate, usable and scalable solutions to the challenge, e.g., Kleinberg et al. (2018). The method holds the hope of being applicable in an even wider range of situations, including the detection of malign intent among people in crowds or queues.

The advent of massive computing power has opened new avenues for establishing reliable methods for detecting deception. Automated techniques offer the hope that huge numbers of statements can be processed efficiently for truth, for example at airports, in a way that the active interviewer approach will never be able to do because of how labour-intensive it is. In the realm of verbal assessment, the use of machine learning to analyse verbal statements has led to several promising results. For instance, Pérez-Rosas et al. (2015) reported an algorithm that correctly classified truth and lies around 70% on a corpus of videos from real trials, substantially higher than human judges watching the same clips. Kleinberg and Verschuere (2021) asked a group of participants to generate accounts of the most important thing they were planning to do during the following week. Another group were allocated those same activities and asked to generate false accounts of how they were going to carry out that activity during the following week. On the basis of the verbal context, the machine learning algorithm correctly decided truth/lie in 69% of cases. Interestingly, when human participants were given the opportunity to adjust or overrule the machine’s decision, performance went to chance level: the humans removed the lie signal picked up by the algorithm.

If advances continue at the same rate it may in the not-too-distant future be possible to apply the machine learning approach to verbal deception usefully in the forensic context. The difficulty is that even if one had a programme with a correct detection rate over 90% there would be many false positives that would need to be checked out and eliminated, conceptually the same problem with mass screening programmes in the health sector. In addition, the approach is still of course far removed from being an instantaneous detector of malign intentions which would be such a revolutionary tool in detecting, for example terrorists at airports; the method requires engagement with the person to be checked, their co-operation and the production and analysis of a written statement. It also seems inevitable that people wishing to avoid detection by such systems will to a certain extent be able to ‘game’ them, which may eventually limit their actual practical use in apprehending sophisticated criminals. In addition, it is of the utmost importance for respect for human rights that such algorithms are created to be fair with regard to race, age and gender, among other variables. In summary, the available programmes of automated lie detection are far from generally applicable.

Table 1^2^ presents an overview of the effect sizes for the different approaches to lie detection. While some of the effects would in other contexts typically be classified as ‘large’, recall that the task these techniques are aimed at solving requires more than a statistically reliable difference. The meta-analyses have different inclusion and exclusion criteria, the application of which can also be a subjective exercise. In addition, the analyses reveal differing levels of publication and other biases, and it is also the case that it is not obvious which summary statistic is the fairest to choose for each analysis. In other words, we present the table while acknowledging that summarising complex analyses in such a fashion is not a substitute for a detailed reading of the meta-analyses.

**Table 1:** The effect sizes of lie detection methods.

Lie detection method	Effect size [95% confidence interval]	Source
CBCA	*d* = 1.01 [0.77; 1.25] *g* = 0.74 [0.42; 1.06]	Oberlader et al., 2016, 2020
Verifiability analysis	*d* = 0.49 [0.25; 0.74]	Verschuere et al., 2020
Cognitive approach	62.15% [51.52; 72.23] of truth tellers were correctly classified and 50.34% [42.28; 58.39] of liars	Mac Giolla and Luke, 2020
SUE	*d* = 1.06 [0.70; 1.43] *g* = 1.72 [1.18; 2.25]	Hartwig et al., 2014; Oleszkiewicz and Watson, 2020

## Discussion

There is empirical and logical evidence that the approach that cunningly times the disclosure of information is potentially useful. It is entirely consistent with the current best advice outlined above. The machine learning approach may well produce viable applicable methods in the not-too-distant future. The other methods reviewed do not seem promising. So, there are no reasons to change the best scientific advice on lie detection, and we now consider *why* the intense research effort has not led to nuances or complexities in the best advice.

When one observes significant differences between true and false statements with a method that analyses verbal output, it does not follow that the method is a promising avenue for forensic use. This is because both of the following may simultaneously be true: A method may reliably generate a measurable gap between liars and truth-tellers *and* be an imprecise tool with low reliability, low consistency of use, what we might call the 54% problem.

By what metric *should* one determine whether the effect size of a cue is practically useful? Bond and DePaulo (2006, p. 230) compared the typical effect size in studies of deception detection favourably to those in social psychology, ‘typical detection abilities are larger than 60% of the research phenomena studied by social psychologists’. To be of merit in theoretical work, it may be sufficient to establish a weak causal link, or even just a weak association, between two variables but in the forensic context, with the big potential downside of miscarriages of justice, one must be more cautious about building on a weak effect. For this reason, as pointed out by Kleinberg and Verschuere (2021), even if one accepts them at face value, it is difficult to see how the findings that humans score only slightly above chance at picking out lies *could* successfully be exploited in practical situations. Indeed, the same doubt would exist if the methods scored, say, 80%, which is higher than the most promising methods achieve.

How could a systematic method that has a consistent success rate of say 80% be used by police? A hit using a method with such an error rate is obviously not sufficient by itself to secure a conviction in most jurisdictions so in an individual case it does not seem advisable to employ. On the other hand, might it be used to identify a main suspect from a larger group of potential perpetrators? That would seem to be a possible but relatively restricted and rare domain of applicability.

There is also the problem of quite how a police investigator or other professional attempting to determine the truth would in practice exploit a method’s putative demonstrated ability to pick up weak signals of deception. Take the verifiability analysis, for instance. How many unverifiable details should a suspect produce, or how low a ratio of verifiable to non-verifiable details would he have to show, for a scientist to advise the police that the suspect is lying?

Generally, the field has indeed been cautious about recommending methods be used in practical situations. This is a responsible attitude because the overeager application of an imprecise method into the legal system seems ideal for producing tunnel vision in investigators and eventually miscarriages of justice. Understandably, many studies have attempted to identify methods with a higher percentage correct discrimination than the Bond and DePaulo 54% rule of thumb. However, despite another couple of decades of intense research effort, it seems that there are no methods with wide domains of applicability that reliably score over, or near, what might be regarded as beyond reasonable doubt. It seems that the percentage game needs rethinking. Note that the approach of disclosing evidence late in proceedings (that in our opinion is the most promising of the human-based methods) finesses the percentage question: SUE is set up to facilitate situations where an interviewee attempting to deceive will produce a demonstrable inconsistency with other evidence.

The continued study of methods in the 70% range seems to us to be of primarily academic interest that may well facilitate the development of social psychological theory, but for which it is less clear what could be a navigable route to practical implementation. It is difficult to see how methods with hit rates or effect sizes between truth-teller and liars that are less than huge could be broadly useful, and, as shown, despite much research effort, these methods do not have success rates even close to what can be regarded as beyond reasonable doubt.

The approaches likely to be successful are those that actively trap the person in a contradiction, not ones that give (statistically significant) differences between liars and truth-tellers. Late timing of confrontation of the witness with information the police have is apparently effective, and for obvious reasons. Thus, in our opinion, there are grounds to pursue research on the optimal use of methods constructed like SUE (initial free account, gradual confrontation with evidence).

## Conclusion

Other than SUE, the methods that were deemed promising a decade ago have not delivered on that promise. The field needs to be careful to avoid what Chu and Evans (2021) have called ‘the ossification of canon’ that arises when a field develops a large literature that renders it more difficult to challenge the status quo. To be fair, as already noted, in this field, there generally is not overenthusiastic recommendation of weak methods. Rather here, the ‘canon’ appears to be that good methods are almost within reach, just over the horizon (see, for example Masip, 2017; Vrij et al., 2018). In addition to the ‘common sense’, ‘late disclosure’ methods that already are functional, the only area where we believe this optimism is at all justified is the domain of automated techniques. This is because, unlike the human experimental psychology, they have shown empirical promise and because there have not already been decades of research on it. Tomas et al. (2022) point out that currently machine learning and psychological approaches do not draw enough on the advantages of the other. They describe the need to combine the strengths of algorithmic methods with psychological theory and detail a promising approach for doing so.

Generally, in our opinion, the literature on deception detection by verbal cues is characterised by the use of solid experimental psychology to tackle questions of high practical importance accompanied by an unwillingness fully to accept the results of said solid science. There is also a risk attached to the repeated pronouncement that there is legitimate hope that the field will soon document successful lie detection methods: Such optimism may provide succour to advocates of dubious or outright useless methods that the empirical database cannot lead one to recommend. If even researchers claim year after year, decade after decade, that some form of psychological strategy represents a promising avenue for reliably detecting lies (without the requisite evidence subsequently being forthcoming), then it is surely easier for disreputable actors also to push their clearly wrong-headed methods.

When additional reliable evidence is available, cunning interviewing with late and gradual presentation of the evidence, as in the SUE technique, embodies a scientifically supported common sense approach. With carefully conducted science, there is considerable potential for automated methods to detect lies in verbal material, in the future. Until then, the best general advice from the psychological literature on verbal lie detection remains simply that a person is lying if what they say is inconsistent either with other things that they have said or with other evidence.

## Author Contributions

From a joint idea, TB produced the first draft which SM revised and edited, and the manuscript was finalised together. All authors contributed to the article and approved the submitted version.

## Conflict of Interest

The authors declare that the research was conducted in the absence of any commercial or financial relationships that could be construed as a potential conflict of interest.

## Publisher’s Note

All claims expressed in this article are solely those of the authors and do not necessarily represent those of their affiliated organizations, or those of the publisher, the editors and the reviewers. Any product that may be evaluated in this article, or claim that may be made by its manufacturer, is not guaranteed or endorsed by the publisher.

## Footnotes

^1^The reference they cite is on the ‘own-race’ effect.

^2^The table was suggested by a reviewer.
